# Sarcopenia and Endocrine Ageing: Are They Related?

**DOI:** 10.7759/cureus.28787

**Published:** 2022-09-05

**Authors:** Prishita Gupta, Sunil Kumar

**Affiliations:** 1 Department of Medicine, Jawaharlal Nehru Medical College, Datta Meghe Institute of Medical Sciences, Wardha, IND

**Keywords:** exercise, replacement, igf-1, testosterone, hormones, muscle loss

## Abstract

Sarcopenia is an illness of the elderly defined by a widespread and gradual decline of skeletal muscle mass and function, with the possibility of negative effects such as poor physical performance, decreased quality of life, and death. Sarcopenia has complicated and multiple pathogeneses. The shift in pathways necessary for muscle regeneration, inflammatory process, and protein synthesis appears to be one of the leading causes of loss of strength and muscle due to age. Researchers have discussed the effects of hypothalamic-pituitary dysfunction in this condition. Lifestyle factors like diet and exercise significantly influence body composition, physical function, and metabolic consequences. The effectiveness and tolerability of hormone replacement in treating sarcopenia will be determined through large-scale clinical trials. In this article, we present a summary of our current knowledge regarding the role of the endocrine system in sarcopenia and an overview of hormonal therapy to address endocrine abnormalities.

## Introduction and background

Sarcopenia

Sarcopenia is the involuntary decrease of strength and mass of muscle with ageing. It affects a person's standard of living by lowering one's capacity to carry with routine activities of daily living and growing impairment that could result in a loss of freedom and the requirement for long-term care. Sarcopenia contributes significantly to the pathophysiology of frailty and functional decline associated with ageing [1]. It encompasses a variety of processes (denervation, mitochondrial malfunction, hormonal and inflammatory alterations, and an increased risk of falling) and several effects.

The number and size of muscle fibers decrease with age, because of a complex process including physical activity, dietary consumption, oxidative damage, and hormonal changes. Although it is unclear how each of these elements contributes explicitly, there is growing proof that the disruption of several age-related positive muscle growth regulators is crucial to the development of sarcopenia [2]. Sarcopenia prevalence rises with age and is estimated to be between 5% and 13% in people's 60s and 70s of life [3].

**Figure 1:**
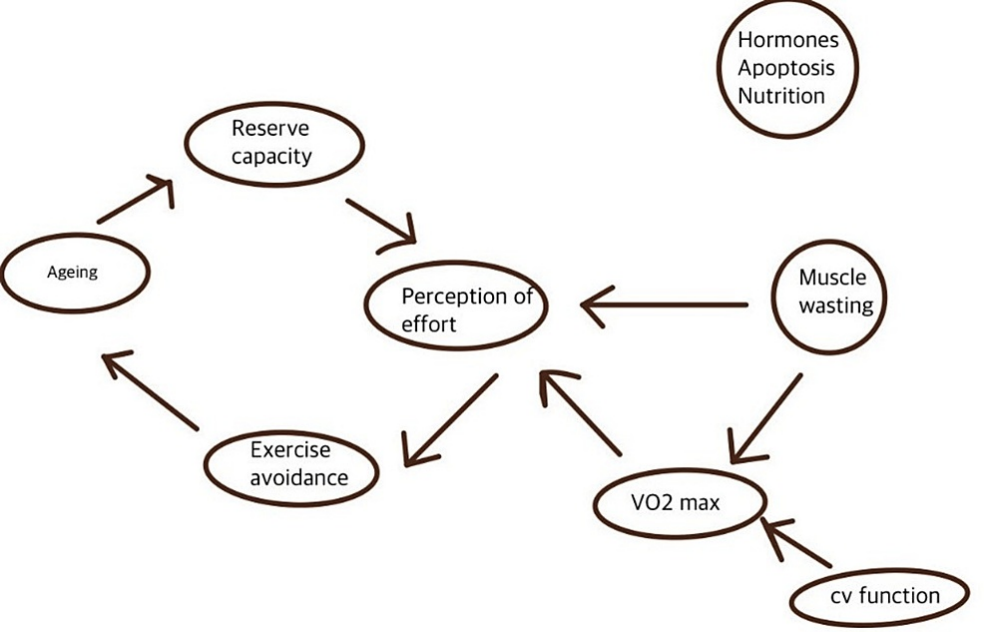
An integrative model showing downregulation of the physiological system in sarcopenia VO2 max: maximum oxygen uptake; CV: cardiovascular This figure is authors' own creation

One of the essential systems in frailty is the endocrine system, which has intricate interactions with the immune system, brain, and skeletal muscle [4]. A decrease in hormones critical for maintaining muscle, such as insulin-like growth factor-1 (IGF-1), testosterone, dehydroepiandrosterone (DHEA), and estrogen, are contributors to sarcopenia. These routes also provide significant intervention prospects [5]. Description of the physiology and downregulation of organ systems in sarcopenia can be seen in Figure 1 [6].

Etiology of sarcopenia

The cause of sarcopenia is unknown; however, some relevant factors have been discovered. Some of them include:

Dietary Factors

Older people tend to reduce their diet significantly. This phenomenon is termed "anorexia of ageing." This can lead to a lack of nutrients, a significant risk factor for sarcopenia [7].

Alcohol and Smoking

Alcohol abusers typically have reduced muscular mass, muscle soreness, cramps, gait issues, and accidents. This refers to as alcoholic myopathy [8]. In diagnostic, in-vivo, and in-vitro research, the impacts of cigarette smoking on muscle metabolism were revealed. Studies discovered structural damage in smokers' skeletal muscle, including the reduced size of type I muscle fibers and smokers' class two fibers [9,10].

Sedentary Lifestyle

Physical activity causes skeletal muscle contraction that enhances energy consumption. Less active older adults are more likely to have decreased muscle mass, putting them in danger of suffering sarcopenia [11].

Endocrine Dysfunction

It is established that sarcopenia is a multifactorial disease. One of the widely discussed causes is endocrine ageing in the elderly. This review focuses on this particular etiology.

Assessment of sarcopenia

The basis of sarcopenia evaluation depends on the mass of muscle, strength, and physical performance.

Assessment of Muscle Strength

The chair stand test and hand grip strength are the most authenticated tools for strength measurement. A dynamometer tests handgrip strength, which corresponds closely with lower-extremity power. Six measurements should be collected, three for each arm. Typically, patients are instructed to squeeze as hard and tight as they can for three to five seconds during each of the six trials; the full measure of the six measurements is generally cited as the final result [12].

The chair stands test measures the strength of quadriceps, hamstrings, glutes, and triceps surae by calculating the time it takes a patient to get up from a sitting posture five times. On the other hand, the timed chair stand test determines how long it takes a patient to rise from a sitting posture in 30 seconds [13].

The one leg standing balance test is also known as the single leg stance time test. Postural balance, a key metric in explaining muscular health, represents a person's capability to sustain postural control in a position, physical movement, and responsiveness to external perturbations without falling [14]. The subject should stand unsupported solely on a single leg and is timed in seconds from when the foot is flexed off the ground to when the standing leg leaves the hips. Participants who cannot sustain the one-leg stance for at least five seconds are considered. Table 1 simplifies the methods and interpretation of the tests performed for sarcopenia.

**Table 1:** Relevant tests and cutoffs for assessment of muscle strength in sarcopenia

TEST	METHOD	CUTOFF
Grip Strength	Dynamometer in hand with the base in the palm - maximal efforts for five seconds	Men: <27 kg Women: <16 kg
Chair Stand test	The time is taken to rise from the seat five times	>15 seconds for five rises
One leg standing test (OSLT)	Amount of time that person can stand on one limb	<5 seconds

Assessment of Muscle Mass

Although widely performed grip strength and gait speed tests remain the most basic and least expensive approaches, muscle mass can be assessed in various ways, most of which use radiological procedures. Magnetic resonance imaging (MRI) and computed tomography (CT) are the gold standards imaging modalities for determining the actual mass of a muscle and its fat infiltration and density [15]. An established technique for assessing frame composition and providing estimations of lean skeletal mass is dual-energy x-ray absorptiometry.

The efficacy of dual-energy x-ray absorptiometry (DXA) for evaluating human muscular tissues and particular pathological circumstances is subject to variation. Additionally, DXA cannot confirm muscular fat, which appears to be becoming more important in terms of the best institutions with positive medical outcomes (compared to CT and MRI) [12].

Assessment of Physical Performance

Physical performance is primarily affected by sarcopenia. Physical performance indicators are essential predictors of impairment among the many variables of functional decline. The short physical performance battery (SPPB) adequately assesses lower extremity physical ability. The SPPB is concerned with three timed tests: the single leg stance time test, the usual gait speed, and the repeated chair stand test [16]. Gait speed over a limited length, usually three to six meters, is a commonly used estimate of objectives performed on older persons. In most cases, the time it takes to walk two or more trials is measured, and the average gait speed over that period is determined and represented in meters per second, or m/s [17].

The endocrine system in the elderly

The endocrine system comprises glands and organs that secrete hormones for various physiological functions. Hormones are chemicals that enhance the functioning of another organ. Hormones, in brief, have a role as messengers, coordinating and directing processes throughout the body. The endocrine system is not immune to the damaging consequences of ageing. Most hormones decline with age; however, some hormones remain stable or increase compared to the young. Although some hormone levels remain consistent, endocrine function depletes typically with age as hormone receptors become less responsive [18].

Ageing is associated with changes in the physique and functional status decline. Healthy older individuals are weaker and have more fat and depleted muscle than younger individuals [19]. The hypothalamus releases hormones that regulate the other endocrine system structures, such as the pituitary gland. Although the quantity of these regulatory hormones remains constant, the action of the endocrine organs can change as we age. Hormones that usually decrease: aldosterone, growth hormone, calcitonin, renin. Hormones that usually increase: norepinephrine, parathyroid hormone, luteinizing hormone, follicle-stimulating hormone. changes in the levels of hormones related to ageing can be seen in Table 2 [20].

**Table 2:** Significant changes in hormones with ageing. IGF-1: insulin like growth factor 1, DHEA-S: dihydroepiandrosterone sulfate

HORMONE	RELATIVE CHANGE WITH AGE
Insulin sensitivity	decreases
Testosterone	decreases
Growth hormone	decreases
IGF-1	decreases
DHEA-S	decreases

Normal circadian rhythms are lost, and hormone secretion declines along most axes. This decline is made worse by reduced tissue sensitivity to hormone activity. These show a decrease in activity in endocrine axes, such as those that affect the adrenal gland, the growth axis, and the reproductive functions (menopause and andropause) (adrenopause) [21].

## Review

Endocrine relation with sarcopenia

The pathogenesis of ageing sarcopenia is unknown; however, the concurrent drop in synthesis rates of muscle proteins and levels of numerous hormones with sarcopenia and their interactions presents an intriguing theory [22]. Hormones play a significant role in muscle development and strength modulation; hence the etiopathogenesis of sarcopenia suggests that the changes in the endocrine system due to ageing can be a possible factor in its development.

**Figure 2:**
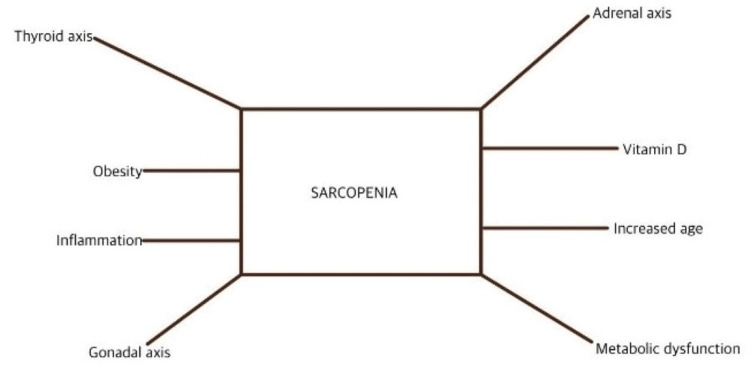
Relationship between hormone dysfunction and sarcopenia This figure is the authors' own creation

A range of chemicals that promote muscle cell growth, such as testosterone, growth hormone (GH), insulin-like growth factor 1, and mechanical growth factor, is reduced after age 60 [6]. Furthermore, there is mounting evidence that the ageing effects of GH, IGF-1, testosterone, and estrogen are linked to the occurrence and pathophysiology of sarcopenia [23,24]. In essence, sarcopenia is a manifestation of widespread endocrine failure. Figure 2 outlines the components involved in these two circumstances, which this paper discusses [25].

Anabolic hormones in sarcopenia

Growth Hormone

The anterior lobe of the pituitary gland secretes GH, also known as somatotropin. Because of this, all tissues can increase the growth rate. Growth hormone synthesis is pulsatile, peaking at the onset of deep sleep and particularly prominent throughout puberty. The growth hormone release is maintained by two hypothalamic hormones, growth hormone releasing hormone (GHRH) and somatostatin. After secretion, it attaches to the pre-dimerized growth hormone receptor (GHR) [26,27]. The activation of GHR helps secrete an additional hormone, insulin-like growth factor-1 (IGF-1).

GH can affect tissues directly through IGF-1 and indirectly. IGF-1 is responsible for around 35% of growth, and GH and IGF-1 working together account for 34% of the shift [28,29]. With incredibly high circulating IGF-1 present during adolescence, GH secretion reaches its peak and gradually declines throughout adulthood [30,31]. GH levels in older persons are lower, and GH pulse amplitude and frequency decrease. Age-related alterations in GH secretion are proposed as potential causes for alterations like decreased muscle mass, muscle strength, in body composition [20].

Skeletal muscle cell protein anabolism levels decline due to variations in GH/IGF-1 levels, which ultimately affect the cells' structure and functionality. As a result, the decrease in GH/IGF-1 contributes to muscle loss [24]. Enhancement in muscle strength and a shift in fiber types have been shown in certain trials of aged participants who receive GH therapy and resistance training [32,33].

Testosterone

Testosterone is the sex hormone and an anabolic steroid in males. Leydig cells secrete testosterone in response to luteinizing hormone (LH) According to several kinds of research, testosterone levels drop by around 1% annually after age 30. As a result, 40% and 70% of men over 70 are likely to have low testosterone levels [34,35]. The combination of the age-associated decline of sex hormones has been shown in numerous epidemiological studies to impact death and osteo-metabolic diseases significantly. Testosterone depletion may adversely affect typical ageing-related differences in body composition and physical function [36]. Satellite cell activation, survival, proliferation, and differentiation are essential for maintaining adult muscle, and testosterone can promote these processes [37]. Table 3 simplifies the mechanism by which testosterone works on the skeletal muscle [38].

**Table 3:** Mechanism of testosterone on skeletal muscle IGF-1: insulin like growth factor 1; MAFbx: muscle-specific ligases atrophy F-box protein

Anabolic	Anticatabolic	Motor neuron
Protein synthesis	Anti-inflammatory	Nerve regeneration
Satellite cells	IGF-1 elevation	Neuritin alpha protein
Insulin sensitivity	Inhibition of MAFbx	

The effectiveness of testosterone was demonstrated in numerous randomized controlled studies (RCTs); however, the outcomes were subject, dose, and treatment method dependent. However, testosterone replacement enhances grip strength and causes adverse effects such as elevated hemoglobin levels. Although a direct comparison of the research is not yet done, the studies suggest that testosterone plays a significant role in establishing and regulating muscle growth and function [39,40].

Estrogen

Estrogen is a sex hormone that has an essential role in a women's reproductive and sexual health. It also affects bones, breasts, skin, heart, pelvic muscles, hair, and other organ systems such as the musculoskeletal system. Menopause is marked by significant hormonal changes, impacting bone mass density and how fat distributes throughout the body. The drop may influence the loss of muscular mass in postmenopausal women in estrogen levels that occur with menopause [41].

Studies have demonstrated that 17-estradiol can particularly suppress the secretion of various pro-inflammatory cytokines during inflammation, such as tumor necrosis factor-alpha (TNF-α), which can break down muscle proteins and impair the muscle's ability to respond to injury [42,43]. A clear relation is found between IL-6 and TNF-α. Estrogen may influence skeletal muscle metabolism directly by activating estrogen receptors found there or indirectly by controlling the GH-IGF-1 axis [25,44]. There have been various studies of hormone replacement therapy approaches. Estradiol-based hormone replacement therapy (HRT) has attracted much attention in some circumstances due to its possible positive effects. Following estradiol hormone therapy, it has been established that menopause-associated obesity and loss of muscle mass can be regained [45].

Ghrelin

A hormone called ghrelin is secreted by enteroendocrine cells in the stomach and other parts of the gastrointestinal system. It is frequently referred to as the "hunger hormone" since it makes people more likely to want to eat. When hungry, ghrelin levels in the blood are at their peak; they then decline after meals. By promoting stomach motility and gastric acid secretion, ghrelin may aid in preparation for meal intake [46,47].

Ghrelin stimulates the release of GH, controls the body's regulatory mechanisms by increasing the sensation of hunger, and promotes obesity through a GH-independent mechanism, among other physiological activities [48]. The ghrelin gene encodes two circulating peptides: acylated ghrelin (AG) and unacylated ghrelin (UnAG). AG promotes hunger, obesity, and a significant release of GH and seems to have an overall anti-inflammatory effect via working through its receptor GHSR1a. Though UnAG does not bind to GHSR1a, it has a similar direct anti-atrophic action to AG on skeletal muscle [49]. The combination of the anabolic effects of ghrelin and its agonists on muscle and appetite make them exciting options for managing cachexia [50].

Catabolic hormones in sarcopenia

Cortisol

The correlation between the hypothalamus, pituitary gland, and adrenal glands refer to as the hypothalamic-pituitary-adrenal axis or HPA axis. The adrenal cortex, which refers to the outer layer of the adrenal glands, receives the hormone adrenocorticotrophic hormone (ACTH) secreted into the blood by the pituitary gland. Adrenal cortex receptors bind to ACTH, which sets off a chain of multiple events that cause the adrenal glands to release glucocorticoids like the hormone cortisol.

Likewise, the HPA axis is known to become dysfunctional with age, which causes the adrenal cortex to release more glucocorticoids [51]. Glucocorticoids in muscle ageing promote the ubiquitin-proteasome and lysosomal systems through enhanced expression of gene atrophy. It also reduces protein synthesis by affecting negative growth regulators like myostatin and anabolic muscle growth factors like IGF-1 [52]. In various population-based studies, short-term glucocorticoids improved muscle strength, function, and physical performance [53].

Myostatin

Myostatin is a strong inhibitor of muscle growth in both humans and animals. It, directly and indirectly, affects the molecular controllers of atrophy and hypertrophy, which may impact physical performance and fitness [54]. Myostatin is a potent inhibitor of muscle growth, a part of the transforming growth factor superfamily. It suggests that myostatin continues to play a function in myogenesis because it is present both prenatally in the growing myotome and postnatally in adult muscles [26,55]. To produce the active C-terminal dimer, furin proteases break the precursor protein of myostatin. Most of the myostatin protein is found in blood, but not all of it appears to be a component of a dormant complex with other proteins, such as propeptide [56].

Tumor Necrosis Factor-alpha

TNF-α is a cytokine with pleiotropic effects on different cell types. TNF-α is primarily released by activated macrophages, T-lymphocytes, and natural killer cells and has a homotrimer protein structure of 157 amino acids. TNF-α may trigger sarcopenia by controlling the quantity or ability of satellite cells to regenerate. Satellite cells are vital myogenic stem cells necessary for muscle growth and regeneration found underneath the basement membranes of muscle fibers [57,58]. Increasing mitochondrial abnormalities, which lead to the loss of muscle fibers, apparently induce apoptosis through upregulated TNF-α in blood and muscle. TNF-α signals trigger apoptosis in muscle [59].

Treatment

There is little evidence that pharmaceutical therapy has any positive effects; thus, resistance training and dietary supplements are the mainstays of the traditional treatment for sarcopenia.

Nutritional Supplements

Nutritional deficit is linked to weakness, making a healthy diet a modifiable risk factor for sarcopenia and a possible target for enhancing physical function in older persons [60]. However, older patients consume less protein than older persons in excellent health, despite substantial evidence relating protein intake to muscle mass. Higher protein intakes (40 g per day) correlated with food supplements and athletic function training in frailer and older people [61,62]. Vitamin D is a fat-soluble vitamin that has the capacity to function as a hormone via a nuclear receptor. The treatment of sarcopenia entails vitamin D administration. The Adequate Intake (AI) for vitamin D is determined by the Dietary Reference Intake (2020), which is 8.5 g/day (340 IU). In two studies, exercise and vitamin D3 supplementation show improvement in physical performance [63,64].

Resistance Exercise

Exercise is a crucial component of treatment plans for sarcopenia because it boosts muscular mass, lowers body fat, and enhances the immune system, cardiovascular system, muscle strength, and endurance. Resistance training is crucial for minimizing wasting. It promotes muscle growth and boosts muscle strength by tipping the scales in favor of synthesis and away from muscle protein breakdown [65]. Muscle quality, force generation capacity, and physical performance are all improved by increased muscle protein synthesis and fiber growth. Progressive resistance training enhances physical performance and peak oxygen uptake [65,66].

Hormone Replacement

There are significant developments that will treat sarcopenia. Selective androgen receptor modulators (SARMs) and growth hormone secretagogues are two prominent endocrine strategies [67]. SARMs work with the androgen receptors in various tissues to deliver a focused, therapeutic impact. They have proven to ameliorate body composition, increase muscle mass and decrease fat composition in various clinical studies [68,69].

One of the hormones that encourage the development of muscular mass is testosterone. It bolsters the skeletal muscle fibers and delays the onset of sarcopenia. But after men turn 30, their typical testosterone levels decline significantly [70]. Lower levels of testosterone may lead to poor clinical outcomes. Since the 1990s, numerous RCTs have investigated the effects of testosterone replacement therapy (TRT) on the elderly. These studies were motivated by the significant functions of testosterone and their lasting beneficial effects on muscle mass and strength in hypogonadal treatment [71]. As part of the natural ageing process, older people notice a decline in their levels of human growth hormone (HGH), but this decline may be reversed if HGH supplements are given to them. The precise mechanism by which HGH repairs and delays the formation of sarcopenia is via activating the IGF-1 molecule [24,72].

Anti-myostatin Therapy

There are several methods for preventing myostatin activity. Adult and older mammals have more skeletal muscle mass when postnatal myostatin inhibition is established. It has been discovered that weekly injections of a myostatin-neutralizing antibody for four weeks dramatically raise the weights of particular muscles by up to 17% [73,74]. Several myostatin inhibitors are now in clinical research for several reasons, primarily cachexia, early postoperative recovery, and sarcopenia. Early clinical trials for muscular dystrophy therapy also test myostatin inhibitors. The discovery of medications to treat muscle wasting has recently focused a lot of interest on the myostatin pathway [75].

## Conclusions

Elderly and non-ageing people with chronic illnesses are frequently affected by sarcopenia, which can harm the course of the disease. There may be no single reason or mechanism for the several physiological and psychological elements that contribute to sarcopenia, which might explain the age-related loss of mass and strength on its own. These factors unfold over a lengthy time. The pathogenesis incorporates many mechanisms unique to each condition that overlap with sarcopenia's ageing process. The overlap between the endocrine dysfunction and the etiopathogenesis of sarcopenia is clear. The endocrine system plays a part in the control of muscle growth, development, and metabolism as hormone secretion changes throughout life. Numerous anabolic hormones and functions likewise decrease with ageing, as does the rate at which critical contractile (myosin heavy chain) and metabolic (mitochondrial) proteins synthesize.

Future studies are necessary to determine whether the advantages of replacing hormones like DHEA and testosterone will be significant enough to outweigh the hazards. Such therapeutic studies ought to use a deterministic methodology. Reduced protein synthesis, a loss of bone and lean body mass, and degradation in immunological function are all effects of the dysfunctional GH-IGF-I axis. Significance of adrenal hormone changes clinically. Resistance training helps delay the adverse effects of sarcopenia, such as muscle mass loss, which frequently causes a decline in strength. Decreasing the effects of sarcopenia should allow for more extended independent living and, when necessary, quicker rehabilitation.
